# A Well-Mixed Computational Model for Estimating Room Air Levels of Selected Constituents from E-Vapor Product Use

**DOI:** 10.3390/ijerph13080828

**Published:** 2016-08-16

**Authors:** Ali A. Rostami, Yezdi B. Pithawalla, Jianmin Liu, Michael J. Oldham, Karl A. Wagner, Kimberly Frost-Pineda, Mohamadi A. Sarkar

**Affiliations:** Research, Development and Regulatory Affairs, Altria Client Services LLC, 601 East Jackson Street, Richmond, VA 23219, USA; yezdi.b.pithawalla@altria.com (Y.B.P.); Jianmin.liu@altria.com (J.L.); Michael.J.Oldham@altria.com (M.J.O.); Karl.a.wagner@altria.com (K.A.W.); Kimberly.frost-pineda@altria.com (K.F.-P.); Mohamadi.a.sarkar@altria.com (M.A.S.)

**Keywords:** e-cigarette, aerosol, e-vapor product, EVP, passive vaping, modeling, computational model, secondhand exposure, exhaled breath, indoor air quality

## Abstract

Concerns have been raised in the literature for the potential of secondhand exposure from e-vapor product (EVP) use. It would be difficult to experimentally determine the impact of various factors on secondhand exposure including, but not limited to, room characteristics (indoor space size, ventilation rate), device specifications (aerosol mass delivery, e-liquid composition), and use behavior (number of users and usage frequency). Therefore, a well-mixed computational model was developed to estimate the indoor levels of constituents from EVPs under a variety of conditions. The model is based on physical and thermodynamic interactions between aerosol, vapor, and air, similar to indoor air models referred to by the Environmental Protection Agency. The model results agree well with measured indoor air levels of nicotine from two sources: smoking machine-generated aerosol and aerosol exhaled from EVP use. Sensitivity analysis indicated that increasing air exchange rate reduces room air level of constituents, as more material is carried away. The effect of the amount of aerosol released into the space due to variability in exhalation was also evaluated. The model can estimate the room air level of constituents as a function of time, which may be used to assess the level of non-user exposure over time.

## 1. Introduction

With the rapid rise in the use of e-vapor products (EVPs), including e-cigarettes and tank devices, public health agencies and U.S. Food and Drug Administration (FDA) have expressed concern about the potential for exposure of non-users to e-cigarette aerosols [1,2]. In 2014 and 2015, FDA Center for Tobacco Products sponsored three public workshops on e-cigarettes. The published proceedings of these workshops called for additional research on exposure and health effects from second- and third-hand exposure to e-cigarette constituents [3]. Second hand aerosol refers to the exhaled aerosol in air and third hand aerosol refers to aerosol deposited on the surfaces in the room. Some of the questions of interest raised at these workshops included (1) How far do aerosols travel in a confined environment? (2) How do exhaled aerosol properties impact second-hand and third-hand exposures, including what chemicals/toxicants are potentially delivered to non-users? (3) What are the potential impacts of e-cigarette use on the levels of particulate matter and chemicals/toxicants in enclosed spaces such as cars, homes, office settings, and public buildings?

Running multiple experimental studies to include all combinations of variables such as indoor space size, air exchange rate, product variation, usage variability, and different e-liquid compositions is a challenge. Computational modeling offers several benefits such as supplementing experimental data, reducing the time and costs of testing, decreasing the need for using human or animal subjects for exposure studies and allowing for efficient estimation of second- and third-hand exposure, under a larger variety of usage and environmental conditions. The US Environmental Protection Agency (EPA) has developed predictive models for environmental assessment, for use under conditions when experimental data is not available or needs to be supplemented. For example, their strategy for assessing chemicals under the Toxic Substances Control Act envisions that predictive tools can be efficiently used in conjunction with experimental assessment methods to help evaluate the fate of chemicals when they are used and released to the environment and how workers, citizens, and the environment might be exposed to and affected by them [4].

Computational models have a long history of use for the purpose of estimating and predicting air quality and the presence of chemicals in indoor environments [5]. Models of both indoor and outdoor air quality assessment have been referenced by the EPA as predictive tools for scientific and educational purposes [6,7,8,9]. Examples of indoor air predictive tools include: IAQX (Indoor Air Quality and Inhalation Exposure) model for multi-zone, multi-pollutant simulations allowing for gas-phase chemical reactions [8], PARAMS 1.0 model for estimating the parameters used in indoor emissions source models, Risk Model to allow for calculation of individual exposure to indoor air pollutants and i-SVOC 1.0 for dynamic modeling of semi-volatile organic compounds in indoor environments. US National Institute of Standards and Technology [9] has introduced a multizone airflow and contaminant transport analysis software (CONTAM) that is widely used for indoor air quality analysis.

Computational and mathematical modeling and simulation of the public health impact of tobacco products has been encouraged for estimating the impact of modified risk tobacco products [10]. Researchers have begun developing mathematical methods and models to make predictions about exposure of e-cigarette aerosol to the users and bystanders. For example, Talih and colleagues [11] created a mathematical model that incorporated design characteristics and user behaviors to predict nicotine delivery. Colard et al. [12] have recently created a physics-based model to predict potential bystander exposure to nicotine. Their model, which incorporated inhalation/exhalation, aerosol propagation/dilution, and air exchange, was used to reproduce a number of results reported in the scientific literature.

A literature survey provided a range of exhaled aerosol properties and room level of constituents from EVP use, which could be used for model inputs and for model validation. Analysis of the exhaled aerosols from e-cigarette use indicated that water and glycerol make more than 99.9% of the constituents [13]. The exhaled aerosol half-life of e-cigarette was found to be approximately 11 s, as compared to a 19–20 min half-life for conventional cigarette smoke [14]. We conducted a controlled clinical study, in which the release of nicotine, propylene glycol, glycerol, and selected toxicants (carbonyls, volatile organic constituents (VOCs), and metals) in exhaled breath air, from the use of three different EVPs was measured [15]. Results showed that on average approximately 3.4%, 6%, and 15% of the inhaled nicotine, propylene glycol, and glycerol were exhaled, respectively. Since exhaled aerosol is the only source of secondhand exposure in the case of EVPs, the exhaled aerosol data collected from this study were used as inputs for our model.

Several studies have reported measurements of room levels of constituents from e-cigarette use in various settings. The most recent article by Hess et al. [16] presented a systematic review of passive exposure to electronic e-cigarette vapor. The authors grouped the studies into four categories involving direct and indirect passive exposure in human volunteers and animals. Results from a majority of the published studies that we reviewed indicate that many chemical constituents (e.g., carbonyls, VOCs, trace metals), with the exception of nicotine, propylene glycol and glycerin, are typically either below levels of detections or present in comparable amounts to those observed under control/background conditions. O’Connell et al. [17] measured the levels of trace metals, tobacco-specific nitrosamines, VOCs, and other constituents in a small meeting room following e-cigarette use. They concluded that exposure of bystanders to the chemicals in the exhaled e-cigarette aerosol, at the levels measured in the study, were below current regulatory standards that are used for workplaces or general indoor air quality. In another experiment [18], glycerol was detected during the e-cigarette vaping session, but nicotine, acrolein, toluene, xylene, nitrogen oxides, carbon monoxide (CO), and polycyclic aromatic hydrocarbons (PAHs) were not detected.

Schober and colleagues [19] reported increases in the room level of 1,2-propandiol, glycerol and nicotine during the vaping session compared with measurements taken on a different control day, with no subject present in the room. Concentration of benzene, acetone, acrolein, and formaldehyde generally did not exceed background levels. The authors reported 30%–90% increases in the sum of 16 PAHs during the vaping sessions as compared with control conditions. However, Farsalinos and Voudris [20] suggested that the differences between control and the vaping sessions could have been in part due to the difference in the level of PAHs present in the environment on the two different testing days. In addition, differences in usage and/or inhalation rates between the vaping sessions, surface deposition, etc. could also account for some of the differences in the results. Schripp et al. [21] also reported slight increases in the amount of aldehydes measured in a test chamber when e-cigarettes were used, compared to when no product was used. They attributed the presence of formaldehyde, acetone, and acetic acid, when no product was used, to the presence of these compounds in human exhaled breath [22,23]. Another study measured indoor air concentrations from e-cigarette use, using validated industrial hygiene sampling methodologies [24]. The study included a large number of participants (*n* = 185 Study 1; *n* = 145 Study 2), and active samples were collected over a 12-h period, for four days. Data from the study also indicated that the majority of chemical constituents sampled were below quantifiable levels of the analytical methods [24].

Data from two studies were used to validate our model. The first set of data came from our controlled clinical study, in which the exhaled aerosol constituents in room air were measured following the use of selected EVPs. In this study, levels of nicotine, propylene glycol, glycerol, 15 carbonyl compounds, 12 volatile organic compounds, and 4 trace metals were measured using ISO or EPA methods [25]. The second dataset is from Czogala et al. [26] who used a smoking machine to generate aerosol. The study measured nicotine, aerosol particle concentration, CO, and VOCs in a chamber where cigarettes and e-cigarettes were used. Nicotine was measurable during the puffing sessions, and was found to be approximately 10 times lower than the levels present during cigarette smoking. The authors concluded that the use of e-cigarettes does not result in significant amounts of VOCs and CO being emitted [26].

We developed and validated a well-mixed computational model that is based on principles similar to those used in the indoor air quality assessment models, referred to by the EPA. The model predicts vapor-particle partitioning and concentration of chemical constituents of aerosol over time, as it travels through a defined indoor space. The model is based on physical and thermodynamic interactions between air, vapor, and particulate phase of the aerosol. These processes are mathematically represented by a set of simultaneous equations including conservation of mass, vapor/liquid partitioning, air flow and species transport, and mixing processes. A number of sensitivity analyses have been performed to evaluate the impact of various parameters that affect the indoor concentration of exhaled aerosol and will be discussed in the paper, along with details on model development and validation.

## 2. Methods

### 2.1. Physical Basis of the Model

The levels of particulate matter and chemical constituents present in a confined space as a result of EVP use depends on (1) the amount of each chemical released into the indoor space upon exhalation by EVP users and (2) dilution of the aerosol due to dispersion and ventilation, as it travels through the confined space. The amount of aerosol and chemicals generated by EVP usage depends on many factors, including liquid composition, device performance, and user behaviors. However, only a fraction of the aerosol inhaled by the EVP user is subsequently exhaled. In addition, the aerosol released into the indoor space undergoes rapid and dynamic change in composition, concentration, and particle size distribution due to dilution by air within the space and ventilation air. The size of the indoor space, the amount and composition of the exhaled aerosol, and the frequency of usage are all important parameters that affect the level of constituents in the indoor space.

The concentration of aerosol that is released into an indoor space rapidly drops as the aerosol is diluted with air. Furthermore, volatile constituents in the aerosol evaporate and result in the shrinking of particle size and changing its composition [14,27]. This phenomenon is easily visible when aerosol from an EVP is exhaled into air as compared with cigarette smoke, which is more stable. The reason for the difference is that most constituents in EVP aerosol are more volatile than constituents in cigarette smoke [28]. As evaporation continues, mixture composition in particles changes, which requires updating the mole fraction of each constituent in order to properly capture the rate of evaporation. This process is transient in time and a vapor-liquid-equilibrium relationship must be used at each time step.

### 2.2. Mathematical Representation

The mathematical representation of a well-mixed model is presented here. Aerosol with a prescribed chemical composition, particle size, and mass density is released into a confined space at a prescribed function of time. The space is ventilated with fresh air at a rate characterized by an air change per hour (ACH). The particles generally shrink due to evaporation of constituents into air, due to dilution. The time scales of interest are much larger than evaporation and mixing times, so that thermodynamic phase equilibrium between the particle and vapor is assumed to hold. Definitions of the terms listed within the equations below are presented in the “Nomenclature” section.

At time *t*, *i* = 1, 2, *…* *N* constituents are present in the space, in the vapor (*v*) and liquid (*l*) phases. The mass balance for each constituent requires:

(1) $$m_{vi}+m_{li}=m_{i}$$

where *m_i_* must be updated for subsequent time steps to account for the mass of this constituent that is released into the space minus that which is carried out by the ventilation air during the corresponding time increment:

(2) $$m_{i}(t+\Delta t)=m_{i}(t)+{\dot{m}}_{in,i}\cdot \Delta t-Q_{a}\cdot \Delta t\cdot C_{i}(t)$$

where *Q_a_* denotes the air ventilation rate. Most constituents enter the space only through the aerosol that is exhaled or machine-generated. However, water enters the equation through multiple sources including: as a constituent in the aerosol, moisture in the air carrying the aerosol, moisture in the room air and in the ventilation air. All sources have been accounted for in the water mass balance. The instantaneous concentration of constituent *i* in the room at time *t* is defined as

(3) $$C_{i}(t)=\frac{m_{i}(t)}{V_{r}}$$

where *V_r_* represents the volume of the indoor space.

The vapor phase concentration (vapor density) of each constituent in air can be expressed as

(4) $$\rho_{vi}=\frac{{\left(\gamma xP_{sat}M\right)}_{i}}{RT}$$

where subscript *i* refers to all variables within the parenthesis in the numerator. It is important to note that at each time step as the concentration of constituent *i* changes due to dilution with incoming air (or moisture in air for the case of water content of a particle), the mole fraction of constituent *i*, *x_i_*, as well as its mass fraction in particle, *y_i_*, will also change. These two are related through mixture relation:

(5) $$y_{i}=\frac{x_{i}M_{i}}{\sum x_{i}M_{i}}$$

Another thermodynamic relationship that will be used is the molecular mass of the mixture in particles, which also varies with time. In terms of the individual mole fractions and molecular mass, it is expressed as:

(6) $$M=\sum x_{i}M_{i}$$

Using these relationships for each of *N* constituents, rearranging, and combining some of those we arrive at *N* simultaneous algebraic equations with *N* unknowns:

(7) $$f(i)=m_{i}w_{i}^{2}+w_{i}(a_{i}M+\sum_{j}m_{j}w_{j}-m_{i})-\sum_{j}m_{j}w_{j}=0$$

with

(8) $$a_{i}=\frac{{\left(\gamma P_{sat}\right)}_{i}V_{r}}{RT}$$

The summation in Equation (7) over j includes all constituents except *i*, that is *j* = 1, 2, *… i* − 1, *i* + 1, *… N*. The new variable *w_i_* is defined as the ratio of mass of *i* in liquid to the total mass of *i* in the space at time *t*:

(9) $$w_{i}=\frac{m_{li}}{m_{i}}$$

Equation (7) was simultaneously solved for *w_i_* for *N* constituents, at every time step, while using previous equations as needed. Initially all *m_i_* as well as the liquid vapor partitioning (*m_li_* (*t* = 0) and *m_vi_* (*t* = 0)) are assumed to be known. An iterative method has been used for each time step until all conditions above, including Equation (6), are met. The *fsolve* function in Matlab^®^ (The MathWorks Inc., Natick, MA, USA) was used in the following examples to solve the system of equations.

### 2.3. Input Variables

Four categories of input data were needed to run the model: indoor space size and ventilation rate, air temperature and humidity, properties and rate of aerosol released into the indoor space, and thermodynamic properties of the constituents of interest.

The indoor space size and ventilation rate greatly affect the concentration of constituents. The dimensions of the space (volume) and ACH were also required to run the model. If ventilation included fresh air as well as recirculated air for humidity and temperature control, the volume of ducts carrying the recirculated were included in the space volume, but only fresh air was included in the ventilation rate. Temperature within the space is also an important parameter in vapor-liquid partitioning and was included in the input data. The aerosol temperature at the time of release into the space is a relevant parameter that defines the vapor-liquid partitioning of each constituent entering the space. However, it is reasonable to assume that the air temperature is not affected by the aerosol temperature as the aerosol mass is significantly less than the air mass of the indoor space.

Properties of aerosol released into the space included aerosol mass, particle size, composition, and the amount of each constituent in vapor and particulate phase. The exhaled aerosol mass, for a given EVP, is highly variable, depending on the vaping habit of the EVP user. For example, some users tend to inhale deeply, while others prefer to exhale after a brief mouth hold. More exhaled aerosol will enter the space in the latter case. The composition of the exhaled aerosol is considerably different from that of the e-liquid in the device. Constituents with high vapor pressure and higher water solubility tend to be absorbed more in the respiratory tract, whereas a higher percent of the inhaled aerosol will be exhaled for the less volatile constituents. The aerosol, depending on the composition can absorb a substantial amount of water during the inhale-exhale process due to the high moisture content of the airways in the lung. Therefore the exhaled aerosol consists of a significant amount of water [13].

Measuring the exhaled aerosol mass and composition released into a confined space during EVP use is a challenging task. Exhaled aerosol properties can be measured by collecting and analyzing the exhaled breath condensate (EBC). However, it is not clear that EVP users exhale in the indoor space the same way that they exhaled into an EBC system. Furthermore, there is substantial inherent variability in how individual users inhale and exhale. Given these challenges, it is generally preferred to validate the model with experimental data in which the aerosol is released into the space at a rate that is controlled. This can be accomplished by generating aerosols using a smoking machine, which has a defined puff duration and puff rate, and directly releasing the aerosol into the space [26]. The level of constituents measured in the space can then be used to validate the model. Once the model is validated, it can be used to estimate the concentration of constituents in an indoor space where EVPs are being used, under a variety of usage and environmental conditions.

The last set of input data for the model was the thermodynamic properties of constituents present in the aerosol, including water, which, because of abundance in air, plays an important role in the final composition. These properties included vapor pressure of each constituent as a function of temperature, activity coefficient, molecular weight, and relative humidity of the indoor space and ventilation air.

## 3. Results and Discussion

Results from two aerosol sources are presented here: (1) smoking-machine generated aerosol and (2) exhaled aerosol released into the indoor space. The concentration of nicotine in the indoor space was estimated using the model and compared with experimental data. Predicted results for glycerol and propylene glycol are also presented. The input data for the two cases were obtained from two separate studies. The data from Czogala et al. [26] were used for the case involving aerosol generated by smoking machines. Although the dispersion of aerosol generated by a smoking machine is not related to the second hand exposure, nevertheless Czogala et al.’s [26] data were used for model validation because of different ventilation rates and aerosol release rates used in the experiment. For the exhaled aerosol case, data from our controlled clinical study were used [25,28].

### 3.1. Smoking Machine-Generated Aerosol Source

Czogala et al. [26] used a smoking machine to produce and release aerosol into a ventilated room that measured 39 m^3^. During a 60 min test, aerosols were released twice at time zero and at 30 min. Each release was either at what they defined as a high (15 puffs) or a low (7 puffs) level. The puff duration and volume were 1.8 s and 70 mL, respectively, with a frequency of one puff every 10 s. Three commercial e-cigarettes with two nicotine levels (1.1% and 1.8%–1.9%) were used in their experiments. Two ventilation levels, as described in [26] (approximately 7 and 10 ACH) were used. Overall, 12 combinations of release level, ventilation level, and e-cigarette were tested. Samples from the room were collected over 60 min and analyzed for CO and nicotine.

The data from four runs using one e-cigarette (EC2) from Czogala et al. [26] study were used for our modeling examples. Some of the input data are shown in Table 1. The aerosol mass delivery per puff was obtained from Goniewicz et al. [29], and the aerosol composition from the smoking machine was assumed to be the same as the e-liquid in the device.

**Figure 1:**
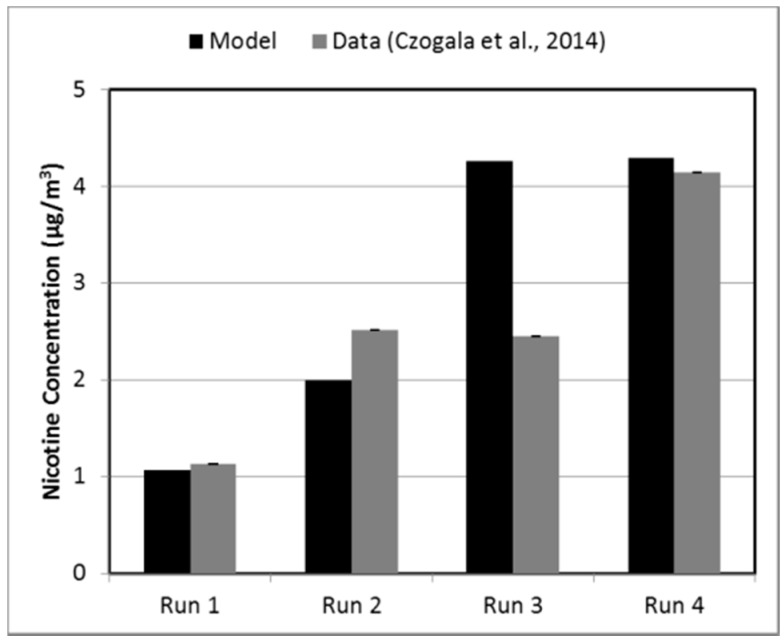
Model prediction and experimental data of average nicotine concentration in the indoor space for smoking machine-generated aerosol source.

The average value of nicotine concentration over 60 min for the four cases as estimated by the computational model, as well as the mean measured data, are shown in Figure 1 along with mean values for these cases. The results may be interpreted using the conditions in Table 1. All conditions were similar during Runs 1 and 2, except for the ventilation level, which was lower for Run 2. At lower ACH, less aerosol was transferred out of the room during the run, and more remained in the room. This can be seen in Figure 1. During Runs 2 and 3, all conditions were almost identical except for the amount of aerosol released into the room, which was higher during Run 3. Figure 1 shows higher predicted nicotine concentration in the room for Run 3. Finally, the conditions for Runs 3 and 4 are very similar, which resulted in similar predicted nicotine concentrations, as shown in Figure 1.

The biggest individual difference was found for Run 3, in which the measured nicotine concentration value was lower than predicted. It was expected that the measured values would be similar for Runs 3 and 4, as all the influencing parameters are approximately the same. It is also worth noting that the sampling point in the room was about 1 m from the e-cigarette location [26], whereas the model results were for the room average values. In addition to the location of the sampling point, other factors might account for the difference in the measured values, including the turbulence in the room which is inherently unsteady, the sample collection method, and variations in sample chemical analysis.

Furthermore, a statistical analysis was performed to determine if the indoor air nicotine levels estimated by the model differed from the experimental data. Given that the data do not follow a normal distribution, the Wilcoxon two-sample test, a nonparametric test, was conducted for this comparison. Significance level was set at *p*-value < 0.05. The results suggested that there is no statistically significant difference between the mean nicotine concentration levels produced by the model and the experiment (*z* = −0.1443, *p* = 0.8852) across the four runs.

**Figure 2:**
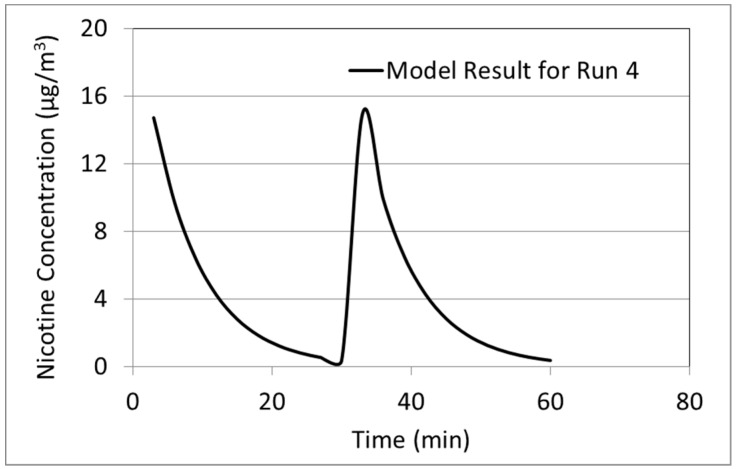
Modeling result for nicotine concentration in the room over time for Run 4 of study by Czogala et al. [26].

A useful result from the model, which is difficult to obtain experimentally, is the evolution of the concentration of constituents in the room over time. This is particularly important if the aerosol release is highly variable in time. An example for nicotine concentration is shown in Figure 2. As expected, immediately after each aerosol release into the room, the predicted average nicotine concentration in the room is the highest. It drops over time as nicotine is carried out by ventilation air. Transient experimental data was not available for this run.

### 3.2. Exhaled Aerosol Source

**Figure 3:**
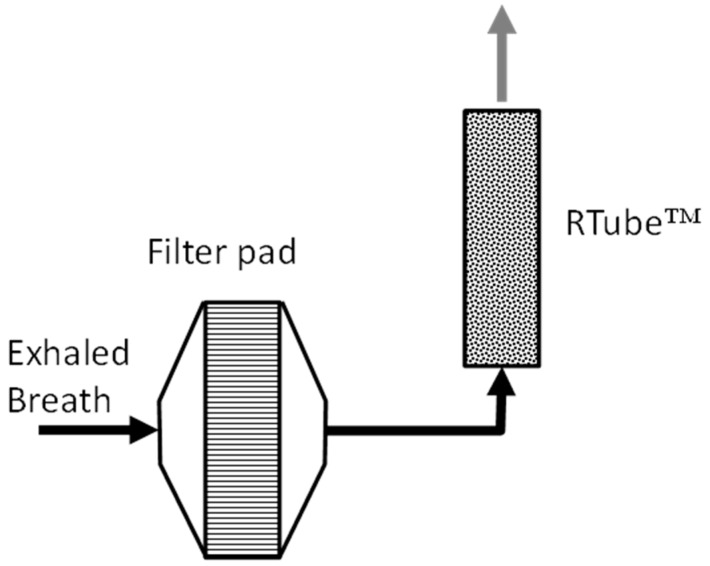
Exhaled breath condensate collection system (EBS) diagram.

Results from the computational model were then compared with the measured data from our internal clinical study. In this study, exhaled breath measurements were made by asking each of 9 study participants to take 10 puffs of an EVP (5 s duration) and after each puff, directly exhale into an exhaled breath system (EBS) shown in Figure 3 [25]. The EBS consisted of a filter and a cryogenically cooled trap. The collected EBS samples were analyzed for nicotine and other constituents. The e-vapor device used for this experiment was a prototype EVP. The e-liquid composition used in the EVP on a weight basis was approximately 41/42/14.6/2.4 of propylene glycol/glycerol/water/nicotine, respectively. The 10-puff average of machine-delivered aerosol mass for 5 s puff with 55 mL puff volume was measured to be 5.2 mg/puff. The amount inhaled by each study participant was assumed to be the same (5.2 mg/puff).

**Figure 4:**
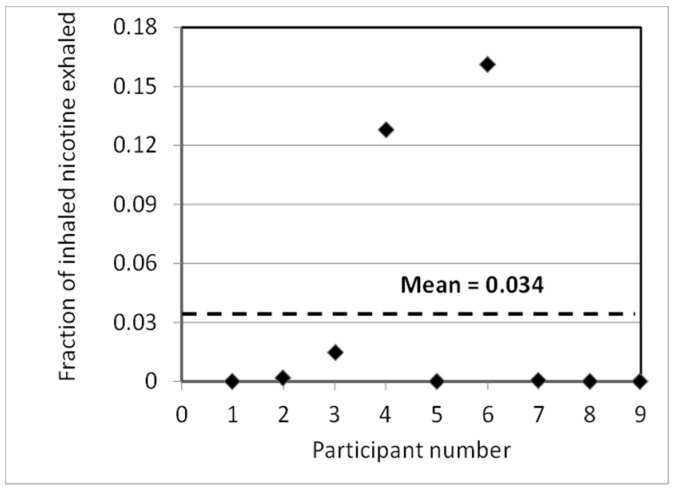
Fraction of inhaled nicotine that is exhaled [27].

The exhaled breath results for nicotine are shown in Figure 4. The *y*-axis represents the fraction of inhaled nicotine that is exhaled. The inhaled nicotine amount is assumed to be 2.4% of 5.2 mg/puff, as described above. Figure 4 shows that there is variability in the exhaled fraction of nicotine among individuals, which may be driven by variability in the individual usage behaviors and depth of inhalation. On average, 3.4% of the inhaled nicotine is exhaled, with 7 of 9 participants exhaling less than 3.4%. Since the same participants used the same EVP in the room air level measurement study, the total exhaled constituents were used as input datum for the computational model to predict the indoor air concentrations.

**Figure 5:**
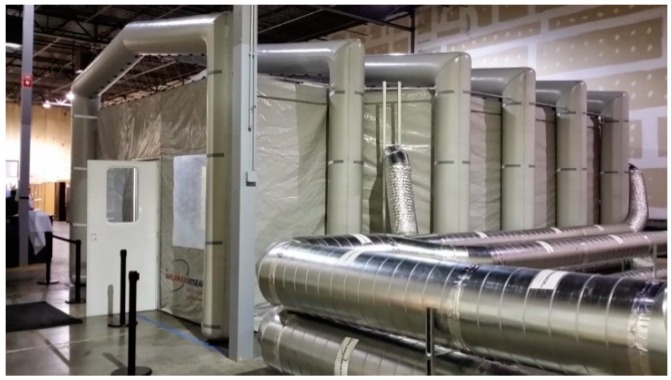
Mobile environmental exposure chamber (mEEC) used for the controlled clinical study.

The controlled clinical study was conducted in a mobile environmental exposure chamber (mEEC) (Inflamax Research, Mississauga, ON, Canada), as shown in Figure 5. The 113 m^3^ mEEC was ventilated and conditioned for temperature. The air circulation rate was 1190 m^3^/h, of which 255 m^3^/h was fresh air that was mixed with the recirculated air. The ACH, based on the fresh air, was calculated to be 2.25 h^−1^. Only the fresh-air rate was used for computational purposes, as the recirculated air does not have any significant effect on the total concentration of the constituents in the mEEC, other than contributing to a better mixing of air in the exposure chamber.

In one study [30], the same 9 participants, whose exhaled breaths were measured earlier, spent 4 h in the mEEC. Each participant was instructed to take 10 puffs, 5 s duration, every 30 min from the same EVP described earlier. Room air samples were collected at six different locations inside the exposure chamber and in the air return line to provide an estimate of the average concentrations of constituents. Indoor air samples were collected over the 4 h duration and analyzed for nicotine and other constituents.

**Figure 6:**
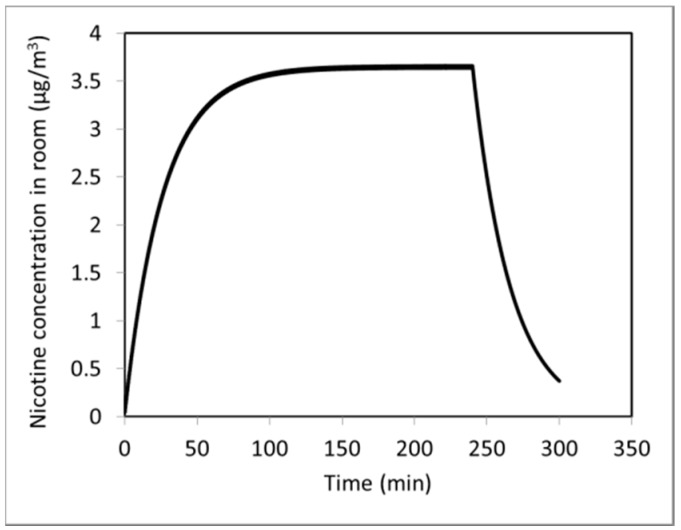
Computational results for nicotine concentration in the mEEC under the described study conditions.

In order to model this case, certain assumptions were made. The main assumptions were (1) participants exhale in the mEEC the same way as they exhaled in the exhaled breath study; (2) 90 puffs (9 participants, each taking 10 puffs) are distributed evenly over each 30 min of mEEC study. Both assumptions impose certain limitations on the accuracy of the model predictions. For example, the back pressure during exhalation into exhaled breath system causes the amount of exhaled aerosol to be different from the exhaled aerosol during normal use of e-vapor in the mEEC. Furthermore, there are puff by puff variation in the exhaled aerosol from an individual user and even more variability among users. Figure 6 shows the predicted concentration of nicotine in the mEEC under the conditions described earlier. For this modeled scenario, nicotine concentration rose over time and reached an equilibrium value of slightly over 3.5 µg/m^3^ after about 100 min. After that, as long as EVP was being used at the same rate, the amount of aerosol emitted into the exposure chamber was balanced by the amount carried out by the ventilation air, and the concentration in the exposure chamber remained unchanged. Once the EVP use was stopped at 4 h, nicotine levels in the room declined rapidly within 1 h.

**Figure 7:**
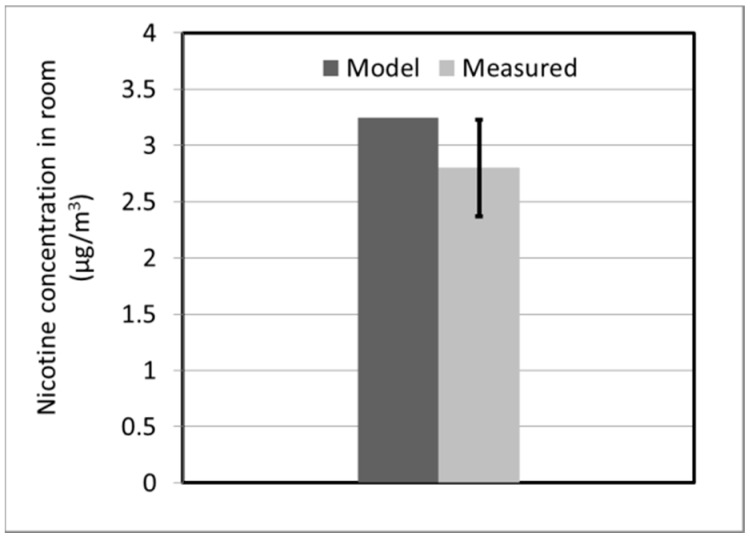
Model predictions compared with measured nicotine concentration in the exposure chamber.

Figure 7 compares the computational predictions with measured value of the average nicotine concentration in the exposure chamber over a 4 h period. The error bar on the experimental data corresponds to the standard deviation of the mean of three replicate runs. Despite the limiting assumptions used in the model development, the prediction is within the range of experimental variability. It is important to note that both the modeled and experimental values are extremely low, and are below the detection limit of method recommended by National Institute of Occupational Safety and Health for indoor nicotine measurement, which is 15 µg/m^3^ (our limit of detection for measurement was 0.25 µg/m^3^). They are also well below the US Department of Labor Occupational Safety and Health Administration permissible exposure limit of 500 µg/m^3^ [31].

The well-mixed model introduced here is not capable of answering the specific question stated at the top of the introduction section “how far do aerosols travel in a confined environment?” To answer this question, a CFD-based distributed model is needed. Results from a distributed model will be presented separately [30]. Now that the well-mixed model has been validated to predict the average room level of nicotine over a prescribed period of time, we will use the model to estimate the room level of nicotine, propylene glycol, and glycerol under different hypothetical conditions.

### 3.3. Examples of Sensitivity Analysis

**Figure 8:**
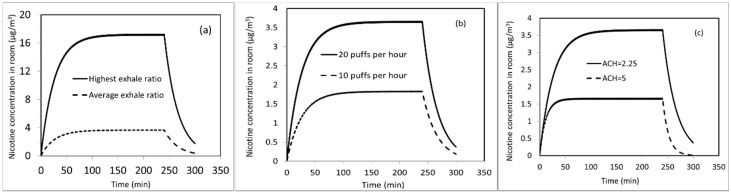
Effects of (**a**) exhaled nicotine ratio; (**b**) number of puffs taken by each participant; (**c**) air exchange rate (ACH) on predicted indoor air nicotine concentration.

After demonstrating that the model can reasonably predict the indoor nicotine concentration under different EVP aerosol source conditions (smoking machine-generated and exhaled aerosols), we used the model to evaluate the effects of different conditions. These analyses are based on the study input data from our controlled clinical study described earlier. In Figure 8a, a hypothetical scenario is considered, where all 9 study participants are assumed to exhale 16% of inhaled nicotine (the highest exhaled % in Figure 4). In the average exhale case, each participant exhaled the average exhale ratio of 3.4%. The results show that predicted equilibrium nicotine concentration after 100 min almost linearly increases with the amount of nicotine exhaled into the exposure chamber.

**Figure 9:**
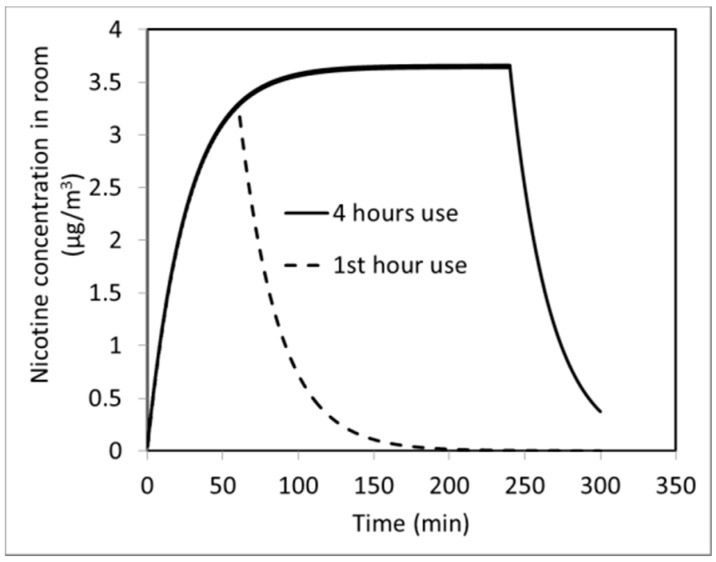
Model predictions for exposure chamber nicotine concentration for 4 h use of e-cigarettes compared with 1 h use.

Figure 8b shows the effect of another usage variability: the number of puffs. Instead of taking 20 puffs/h, it is assumed that participants take 10 puffs/h. As expected, the predicted indoor air nicotine concentration drops almost 50% to a steady-state value of 1.6 µg/m^3^. Figure 8b shows the steady state concentration of nicotine is linearly proportional to the number of puffs; doubling the number of puffs almost doubles the concentration. The effect of air exchange rate is shown in Figure 8c. Increasing the ACH from 2.25 to 5 reduces the predicted concentration proportionally, as the amount of nicotine carried out by the ventilation air increases, with less remaining indoor. As shown in Figure 8c, the predicted steady-state condition is reached in a shorter time (50 min for 5 ACH vs. 90 min for 2.25 ACH). Figure 9 shows predicted exposure chamber concentration of nicotine when the EVP is used only during the first hour of a 4 h period. In this case, the concentration drops exponentially with time, and it takes about 2 h to go back to baseline.

**Figure 10:**
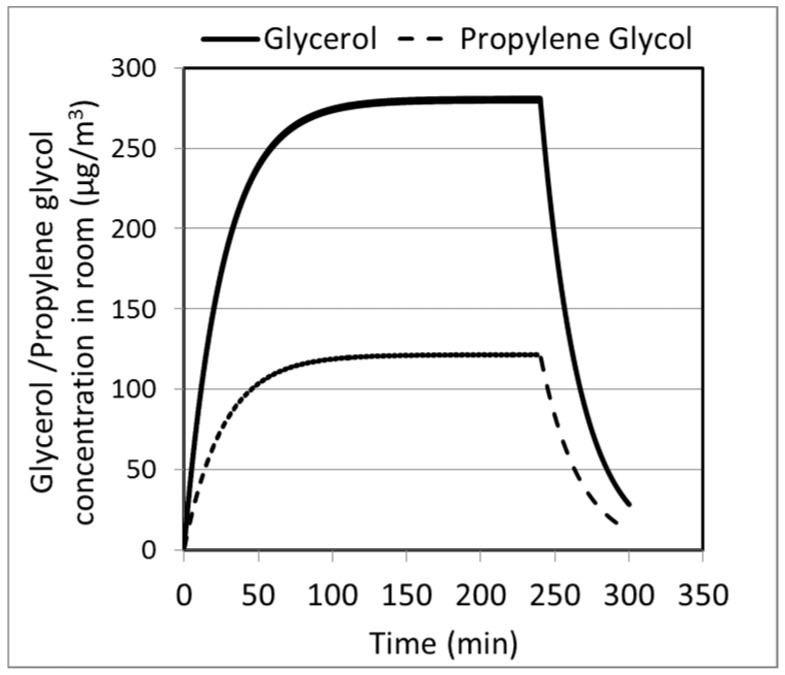
Model predictions for glycerol and propylene glycol concentrations in the mEEC.

The model may be used for prediction of other constituents in room air. Figure 10 shows such predictions for glycerol and propylene glycol levels in mEEC. The transient behaviors are similar to the nicotine concentration, with the higher values attributed to higher concentrations of propylene glycol and glycerol in the e-liquid.

Finally, it is worth pointing out that according to the model predictions, aerosol constituents rapidly evaporate resulting in almost 100% of each constituent in vapor phase. As a result, the particle mean diameter drops from the initial value of 0.5 µm to a nanometer size in a short time. This is consistent with Bertholon et al. [14] and Fernandez et al. [32] measurements that the half-life of exhaled e-cigarette aerosol is short, typically about 10 s, which corresponds to the time steps used in this model calculation. In reality, particles are visible in the vicinity of the exhaled position at short times, but they disappear visibly as they travel farther away from the source and mix with the room air. The spatial variation of particle size and concentration cannot be predicted by the current well-mixed model. That information, along with spatial variation of the room level of constituents, is the subject of a distributed model that can accurately capture the temporal and spatial mixing process. We have also developed a CFD-based distributed model, which will be published separately.

## 4. Conclusions

A well-mixed computational model has been developed using principles similar to those referenced by EPA for indoor air quality analysis where EVPs are used. The mechanistic model is based on physical and thermodynamic interactions between air, vapor, and particles in aerosol. The results from a well-mixed model were presented, and they agree with measured values of nicotine concentration in indoor spaces following the release of aerosols from two different sources: smoking machine-generated and exhaled aerosol. The model introduced in this study can serve as a useful tool to estimate the level of constituents from exhaled EVP aerosols under a wide variety of usage conditions and different types of confined spaces e.g., cars, or large commercial rooms, where accurate measurement is difficult and resource intensive.

## Figures and Tables

**Table 1:** Input data from four runs using one e-cigarette as described in the text.

Run Number ^1^	Nicotine Level in E-Liquid (%)	Aerosol Release	Ventilation Level (ACH) ^2^
1	1.8	Low (7 puffs)	9.86
2	1.8	Low (7 puffs)	6.81
3	1.8	High (15 puffs)	6.83
4	1.8	High (15 puffs)	6.80

^1^ Naming of run numbers is different from Czogala et al. [26]; ^2^ ACH, air change per hour.

## Abbreviations

The following abbreviations are used in this manuscript:

ACH	air change per hour
CFD	computational fluid dynamics
CO	carbon monoxide
EBC	exhaled breath condensate
EBS	exhaled breath system
EPA	US Environmental Protection Agency
EVP	e-vapor product
FDA	U.S. Food and Drug Administration
mEEC	mobile environmental exposure chamber
PAH	polycyclic aromatic hydrocarbons
VOC	volatile organic constituent

## Nomenclature

The following variables are used in this manuscript:

*m_vi_*	mass of vapor of constituent *i* in indoor space at time *t* (kg)
*m_li_*	mass of liquid of constituent *i* in indoor space at time *t* (kg)
*m_i_*	mass of constituent *i* in indoor space at time *t* (kg)
*C_i_*	concentration of constituent *i* in indoor space (kg/m^3^)
*V_r_*	volume of indoor space (m^3^)
*Q_a_*	air ventilation (m^3^/s)
*t*	time (s)
*γ_i_*	activity coefficient (dimensionless)
*x_i_*	mole fraction of *i* in particle, liquid phase
*y_i_*	mass fraction of *i* in particle, liquid phase
*P_sat_*	saturation pressure at given temperature (kPa)
*R*	universal gas constant (kJ/(kmol·K))
*T*	temperature (K)
*M_i_*	molecular mass of *i* (kg/kmol)
*M*	molecular mass of the mixture in particle (liquid phase) (kg/kmol)
ρ	density of vapor of *i* in air (kg/m^3^)
